# Supplementary material for: Atg1 kinase regulates autophagosome‐vacuole fusion by controlling SNARE bundling
Source: EMBO Rep. 2020 Dec 3;21(12):e51869. doi: 10.15252/embr.202051869 (PMC7726815; doi:10.15252/embr.202051869)
Supplement: Supplementary file 1 — Appendix [file EMBR-21-e51869-s001.pdf]

Appendix

Atg1 kinase regulates autophagosome-vacuole fusion by controlling SNARE bundling

Table of contents

Appendix Figure S1: Rescue analysis of YKT6 mutants	page 2
Appendix Table S1 - Yeast strains used in this study	page 4
Appendix Table S2 - Plasmids used in this study	page 4
Appendix References	page 5

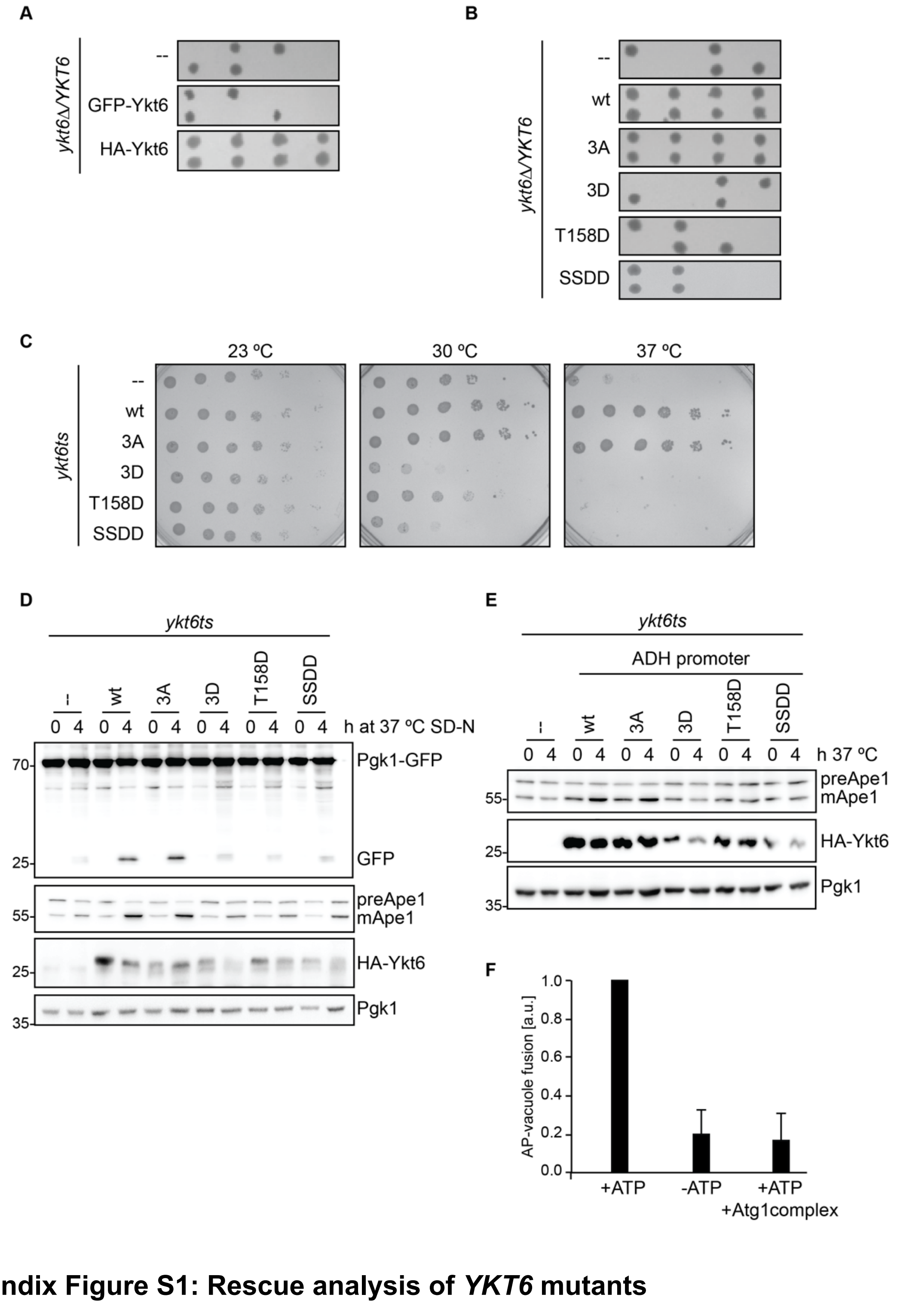

Appendix Figure S1: Rescue analysis of *YKT6* mutants

A) Heterozygous diploid knock-out *ykt6Δ*/*YKT6* cells containing an empty plasmid, a centromeric GFP-Ykt6 or a 3HA-Ykt6 plasmid as indicated were sporulated, and tetrads were dissected on YPD plates followed by growth at 30 °C. Two tetrads are shown for each strain and distributed from left to right. Complementation of the *ykt6Δ* results in the growth of all four spores.

B) Heterozygous diploid knock-out *ykt6Δ/YKT6* cells containing an empty plasmid, 3HA-Ykt6 wild type, 3HA-Ykt6-3A, 3HA-Ykt6-3D, 3HA-Ykt6-T158D or 3HA-Ykt6-SSDD were sporulated, and tetrad dissection analysis was performed as in A).

C) The *ykt6ts* strain was transformed with an empty plasmid, 3HA-Ykt6 or 3HA-Ykt6 mutant variants as indicated. Transformants were grown to late exponential phase at the permissive temperature (23 ºC). Cells were diluted to OD600 0.4 and spotted in 5-fold serial dilutions on selective media plates. Plates were incubated at permissive (23 ºC) or restrictive temperature (30 ºC and 37 ºC) as indicated. One representative experiment out of three biological replicates is shown.

D) *ykt6ts* Pgk1-GFP cells containing an empty plasmid or 3HA-Ykt6 or 3HA-Ykt6 mutant variants were grown at permissive temperature (23 ºC) to late exponential phase in selective medium and shifted to the restrictive temperature at 37 °C for 1 hour (time point 0 h), followed by 4 hours starvation in SD-N medium at restrictive temperature (37 ºC) (time point 4 h). TCA extracts were prepared and analyzed by anti-GFP, anti-Ape1, anti-HA and anti-Pgk1 Western blotting. One representative experiment out of three biological replicates is shown.

E) *ykt6ts* cells containing an empty plasmid, 3HA-Ykt6 or indicated mutants thereof expressed under the ADH1 promoter were grown at permissive temperature (23 ºC) to late exponential phase in selective medium. Cells were shifted for 1 hour to the restrictive temperature at 37 °C (time point 0 h) and continued to grow at restrictive temperature (37 ºC) for 4 hours more (time point 4 h). TCA extracts were prepared and analyzed by anti-Ape1, anti-HA and anti-Pgk1 Western blotting. One representative experiment out of three biological replicates is shown.

F) Autophagosomes were prepared from GFP-Atg8 *vam3Δ pep4Δ* cells starved for 16 hours at 23 °C. Vacuoles were isolated from Vph1-4xmCherry *atg15Δ* pep4Δ cells grown under rich conditions at 30 °C. Fusion reactions were incubated at 30 °C for 2 hours with or without the addition of ATP and yeast purified Atg1 kinase complexes as indicated. The mean from three independent biological experiments is shown. Error bars represent standard deviation.

Appendix Table S1 - Yeast strains used in this study

Name	Genotype	Background	Source
BY4741	his3Δ1 leu2Δ0 met15Δ0 ura3Δ0; Mat a	BY474x	Euroscarf
yCK660	atg1::G418	BY474x	Euroscarf
yCK801	PHO8::PHO8Δ60-HIS	BY474x	(Bas et al. 2018)
yCK858	ATG1-TAP:HIS ATG29-GFP:HIS	BY474x	This study
yFK7	Vph1-4xmCherry:URA3 pep4::NAT atg15::G418	BY474x	(Hollenstein et al. 2019)
yFK28	sfGFP-ATG8 ykt6ts:G418 vam3::G418	BY474x	This study
yFK79	sfGFP-ATG8 atg1::G418 vam3::G418	BY474x	This study
yFK80	sfGFP-ATG8 ykt6ts:G418	BY474x	This study
yLB180	sfGFP-ATG8 vam3::G418	BY474x	(Bas et al. 2018)
yLB189	sfGFP-ATG8 pep4::NAT vam3::G418	BY474x	(Bas et al. 2018)
yLB209	sfGFP-ATG8 atg1::G418	BY474x	This study
yLB224	sfGFP-ATG8 pep4::NAT vam3::HPH ykt6ts:G418	BY474x	(Bas et al. 2018)
yLB253	YKT6/ykt6::G418	BY4743	Euroscarf
yRT186	PHO8::PHO8Δ60-HIS3 ykt6ts:G418	BY474x	(Bas et al. 2018)
yTB282	sfGFP-ATG8	BY474x	This study

Appendix Table S2 - Plasmids used in this study

Name	Characteristics	Promoter	Terminator	Source
pRS315	CEN, LEU2	-	-	(Sikorski & Hieter 1989)
pRS316	CEN, URA3	-	-	(Sikorski & Hieter 1989)
pRS416	CEN, URA3	-	-	(Sikorski & Hieter 1989)
pRS415	CEN, LEU2	-	-	(Sikorski & Hieter 1989)
pGEX4T1		tac	-	GE Healthcare
pGEX5x2		tac	-	GE Healthcare
pDP299	GFP-Ykt6, pRS415	YKT6	CYC1	This study
pFK33	3HA-Ykt6, pRS415	YKT6	CYC1	This study
pFK34	3HA-Ykt6, pRS416	YKT6	YKT6	This study
pFK35	3HA-Ykt6 (T158A, S182A, S183A), pRS416	YKT6	YKT6	This study
pFK36	3HA-Ykt6 (T158D, S182D, S183D), pRS416	YKT6	YKT6	This study
pFK38	3HA-Ykt6 (T158D), pRS416	YKT6	YKT6	This study
pFK40	3HA-Ykt6 (S182D, S183D), pRS416	YKT6	YKT6	This study
pFK47	GST-Ykt6 (1-194), pGEX4T1; [Ykt6Δac]	tac	-	This study
pFK48	GST-Ykt6 (1-194, T158A; S182A; S183A), pGEX4T1; [Ykt6Δac, A mutation]	tac	-	This study
pFK49	GST-Ykt6 (142-194), pGEX4T1; [SNARE Ykt6Δac]	tac	-	This study
pFK50	GST-Ykt6 (142-194, T158A, S182A, S183A), pGEX4T1; [SNARE Ykt6Δac; A mutation]	tac	-	This study
pFK51	GST-Ykt6 T158 peptide (150-166), pGEX4T1	tac	-	This study
pFK52	GST-Ykt6 T158A peptide (150-166), pGEX4T1	tac	-	This study
pFK53	GST-Ykt6 S182S183 peptide (175-190), pGEX4T1	tac	-	This study
pFK54	GST-Ykt6 S182AS183A peptide (175-190), pGEX4T1	tac	-	This study
pFK58	3HA-Ykt6, pRS416	ADH1	YKT6	This study
pFK59	3HA-Ykt6 (T158A, S182A, S183A), pRS416	ADH1	YKT6	This study
pFK60	3HA-Ykt6 (T158D, S182D, S183D), pRS416	ADH1	YKT6	This study
pFK61	3HA-Ykt6 (T158D), pRS416	ADH1	YKT6	This study
pFK62	3HA-Ykt6 (S182D, S183D), pRS416	ADH1	YKT6	This study
pSR53	Pgk1-GFP, pRS315	PGK1	ADH1	This study

Appendix References

Bas L, Papinski D, Licheva M, Torggler R, Rohringer S, Schuschnig M & Kraft C (2018) Reconstitution reveals Ykt6 as the autophagosomal SNARE in autophagosome-vacuole fusion. *J Cell Biol* 217: 3656–3669

Hollenstein DM, Gómez-Sánchez R, Ciftci A, Kriegenburg F, Mari M, Torggler R, Licheva M, Reggiori F & Kraft C (2019) Vac8 spatially confines autophagosome formation at the vacuole. *Journal of Cell Science*: jcs.235002–25

Sikorski RS & Hieter P (1989) A system of shuttle vectors and yeast host strains designed for efficient manipulation of DNA in Saccharomyces cerevisiae. *Genetics* 122: 19–27
